# Infant Motor Development Predicts Sports Participation at Age 14 Years: Northern Finland Birth Cohort of 1966

**DOI:** 10.1371/journal.pone.0006837

**Published:** 2009-08-31

**Authors:** Charlotte L. Ridgway, Ken K. Ong, Tuija H. Tammelin, Stephen Sharp, Ulf Ekelund, Marjo-Riitta Jarvelin

**Affiliations:** 1 MRC Epidemiology Unit, Institute of Metabolic Science, Cambridge, United Kingdom; 2 Finnish Institute of Occupational Health, Oulu, Finland; 3 Imperial College London, London, United Kingdom; 4 Institute of Health Sciences, University of Oulu, Oulu, Finland; Universidad Europea de Madrid, Spain

## Abstract

**Background:**

Motor proficiency is positively associated with physical activity levels. The aim of this study is to investigate associations between the timing of infant motor development and subsequent sports participation during adolescence.

**Methods:**

Prospective observational study. The study population consisted of 9,009 individuals from the Northern Finland Birth Cohort 1966. Motor development was assessed by parental report at age 1 year, using age at walking with support and age at standing unaided. At follow up aged 14 years, data were collected on the school grade awarded for physical education (PE). Self report was used to collect information on the frequency of sports participation and number of different sports reported.

**Principal Findings:**

Earlier infant motor development was associated with improved school PE grade, for age at walking supported (p<0.001) and standing unaided (p = <0.001). Earlier infant motor development, in terms of age at walking supported, was positively associated with the number of different sports reported (p = 0.003) and with a greater frequency of sports participation (p = 0.043). These associations were independent of gestational age and birth weight, as well as father's social class and body mass index at age 14 years.

**Conclusions:**

Earlier infant motor development may predict higher levels of physical activity as indicated by higher school PE grade, participation in a greater number of different types of sports and increased frequency of sports participation. Identification of young children with slower motor development may allow early targeted interventions to improve motor skills and thereby increase physical activity in later life.

## Introduction

There are a wide range of health benefits associated with being physically active during adolescence, both in terms of physical [1] and mental well being [2] and there is evidence that the protective effects of physical activity are independent of cardio-respiratory fitness and body fat in young people[3].

Previous cross-sectional studies in children and adolescents suggest that motor proficiency is positively associated with physical activity levels [4], [5] and one longitudinal study observed that childhood motor skills predicted fitness in adolescents [6]. Furthermore interventions to improve motor skills not only increased activity levels but improved the reported enjoyment of physical activity [7], suggesting competence or perceived competence may be important in determining physical activity levels [8], [9].

There is also evidence that motor development during infancy may be associated with physical performance later in life. Earlier age at first walking was positively associated with adult physical performance, in terms of chair rising and standing balance at age 53 years [10] and hand grip strength [11]. Previous research within the Northern Finland Birth Cohort of 1966 (NFBC 1966) identified linear associations between infant motor development and adult physical performance in terms of muscle strength, muscle endurance and cardio-respiratory fitness at age 31 years, which was independent of birth weight, infant growth and adult body size [12].

It is recognised that physical activity levels decline during adolescence [13], [14], [15]. Sports participation contributes to higher levels of physical activity [16], [17] and voluntary sports participation could be important for maintaining higher activity levels throughout adolescence [18]. Furthermore, evidence suggests that sports participation during adolescents can predict adulthood physical activity levels [19], potentially influencing health outcomes in later life.

The aim of this present study was to investigate associations between the timing of infant motor development and subsequent sports participation and physical education grade during adolescence in a large prospective population based cohort in Northern Finland. We hypothesised that earlier infant motor development predicts increased sports participation and higher physical education grade at age 14 years.

## Methods

### Ethics Statement

Mothers, in consultation with nurses, agreed to take part in the study prior to the birth of their child. Offspring were followed up at age 14 years and 31 years, and at the latter time point informed written consent was provided for follow-up and for use of previously collected data. The ethics committee of the University of Oulu approved the study.

### Study Population

The study population was comprised of individuals from The Northern Finland Birth Cohort of 1966 (NFBC 1966), who were recruited from all pregnancies with a birth expected between January 1^st^ and December 31^st^ in 1966 within Finland's two most northerly provinces, Oulu and Lapland. This cohort has been described previously [20]. Briefly the cohort consisted of 12,058 births, an estimated 96.3% of all live births during the qualifying period. At age 1 year children were assessed including parental report of the age in months when reaching specific developmental milestones.

A total of 9,842 individuals had complete data for both birth weight and developmental milestone measures at one year. Those with a gestational age of less than 36 weeks were excluded (n = 252) and a small number of children with severe hearing (n = 32) or sight (n = 75) defects were excluded to avoid inclusion of individuals with more severe congenital diseases. A further small number were excluded if the timing of the 1 year visit was carried out at <300 days old (n = 3) or >500 days old (n = 46). This left a dataset of 9,434, of these 9,009 individuals had at least one motor milestone recorded at the time of the one year assessment (age at walking supported n = 8,814 and age at standing unaided n = 7,445) and at least one outcome measure of sports participation or school physical education grade.

### Infant motor development

Infant motor development was assessed at age 1 year by parental report and was defined as 1) age at walking supported; and 2) age at first standing unaided. Information on infant motor development was collected on 91% of all infants, with 95% of those being at least 11.5 months old at the time of the visit.

### Adolescent sports participation

Individuals were followed up at age 14 years, with self report questionnaires being mailed to all subjects with a known address. 11,399 individuals responded (97% response rate). The questionnaire included self report questionnaire on school physical education grade (PE grade) and participation in sports. These questions were used to provide three measures of sports participation: school PE grade, the number of different sports they participated in regularly and finally the frequency of sports participation per week.

The school PE grade across Finland at that time was based on a combination of skills, action and attitude during physical education classes (graded from 4 to 10 being the highest). This grade therefore not only reflects physical performance but also to a certain extent an individual's attitude towards physical activity and sports participation at school. Self report data were available on school sports grade for 6,121 of the individuals included in this study.

At age 14 years self report data were also collected on the different types of sports individuals regularly participated in, categorised into 20 different sports groups, which have previously been described [19]. Briefly commonly reported sports were categorised individually, for example ice hockey and soccer, whereas minority sports were grouped together into groups of similar sports, such as tennis, table tennis and badminton being categorised as ‘racket games’. These data were used to create an ordered categorical variable of ‘number of different sports’. Data on the number of different sports reported were available for 8,998 individuals.

Frequency of sports participation outside of school hours was also reported as 1) ‘less than once a week’, 2) ‘once a week’, 3) ‘twice a week’, 4) ‘every other day’ and 5) ‘every day’ and recoded into frequency per week (1 = 0, 2 = 1, 3 = 2, 4 = 3 and 5 = 7 times per week) Data on reported frequency of sports participation were available for 7,736 individuals.

### Potential confounding variables

Birth weight was measured by midwives and gestational age was recorded, with 99% of births being in hospitals. Height and weight at age 14 years were self reported, from which Body Mass Index (BMI = weight (kg) divided by height (m) squared) was calculated.

Social class data for the father in 1966 (year of birth of children) were collected by a self report questionnaire, as routinely defined in Finland into four categories: 1) I and II skilled professionals. 2) III skilled workers. 3) IV unskilled workers and 4) Farmers (with any farmers of large ranches being classified as class III) [21].

As season of birth has previously been associated with sports performance, possibly because of differences in relative age [22], birth season was categorised into three ordinal groups based on the school calendar, classifying date of birth into three seasons: 1) Spring (January to April) n = 2,651, 2) Summer (May to August) n = 3,031 and 3) Autumn (September to December) n = 2,661.

### Calculations and statistics

Mean and standard deviation (SD) for descriptive variables are displayed along with independent sample t-tests to investigate gender differences in exposure and outcome variables. Internally derived sex specific SD scores were calculated for birth weight and BMI at age 14, by subtracting the sample mean from the individual mean and then by dividing by the sample standard deviation. A change in SD score of 0.67 SD units, would represent the distance between each centile line on a standard growth chart (i.e. 2^nd^, 9^th^, 25^th^, 50^th^, 75^th^, 91^st^, 98^th^ centile lines).

Univariate associations between exposure and outcome variables were examined by simple Pearson's correlations. Multiple linear regression models were performed separately for each measure of infant motor development (i.e. age at walking supported and age at standing unaided) to examine their association with the three outcome variables (i.e. PE grade, frequency of sports participation and number of different types of sports reported). All exposure variables were treated as linear. For illustrative purposes infant motor development variables were categorised into approximate quintiles (<9, 9, 10, 11, or ≥12 months of age).

Initial analyses revealed no evidence for any interactions between sex and infant motor development, so all subsequent analysis was carried out in the whole dataset and multiple linear regression models were adjusted for sex, gestational age, birth season and father's social class category in 1966. We thereafter reanalysed our data using a quadratic term for each of the exposure variables to examine potential non-linear associations. However there was no evidence for any non-linear associations.

Finally, significant associations between motor development and physical activity participation from the linear multiple regression analyses were further adjusted for birth weight and BMI at age 14 years to examine whether the associations observed were independent of birth weight and BMI.

A significance level of 0.05% was used and all statistical analyses were carried out using SPSS version 14.0 (SPSS Inc, US).

## Results

Table 1 displays the descriptive statistics measured at birth, age 1 year and at age 14 years for the study population. Boys achieved walking with support slightly earlier than girls (p = 0.046) but there were no sex differences for age at first standing unaided (p = 0.296).

**Table 1:** Descriptive Statistics.

	All		Male		Female		t-test
	Mean	(SD)	Mean	(SD)	Mean	(SD)	P
Birth weight (kg)	3.53	(0.49)	3.59	(0.50)	3.45	(0.48)	<0.001
Walk supported (months)	9.12	(1.36)	9.10	(1.36)	9.14	(1.37)	0.046
≤6 months (%)	1.6		1.5		1.7		
7 months (%)	10.6		10.6		10.6		
8 months (%)			23.0		22.0		
9 months (%)	22.5		25.0		24.2		
10 months (%)	24.6		23.1		24.5		
11 months (%)	14.1		14.1		14.0		
≥12 months (%)	2.8		2.6		3.0		
Stand unaided (months)	10.21	(1.21)	10.24	(1.22)	10.18	(1.23)	0.286
≤7 months (%)	2.0		1.9		2.1		
8 months (%)	6.6		6.2		7.0		
9 months (%)	17.7		17.1		18.3		
10 months (%)	28.7		29.2		28.2		
11 months (%)	32.7		33.0		32.4		
≥12 months (%)	12.3		12.5		11.8		
**Follow up at 14 years**							
Weight (kg)	51.9	(9.1)	53.0	(10.1)	50.7	(7.9)	<0.001
Height (m)	1.63	(0.08)	1.65	(0.09)	1.62	(0.06)	<0.001
BMI (kg/m^2^)	19.3	(2.5)	19.3	(2.6)	19.4	(2.5)	0.257
School PE grade (range 4 low-10 high)	8.01	(0.87)	7.98	(0.89)	8.04	(0.84)	0.003
Number of different types of sports activities	1.49	(1.16)	1.53	(1.13)	1.44	(1.18)	<0.001
Frequency of sports participation (per week)	2.42	(2.34)	2.88	(2.43)	1.96	(2.16)	<0.001

School sports grade (n = 6,212).

Number of different sports activities reported (n = 8,998).

Frequency of sports participation (n = 7,736).

At age 14 years body weight and height were higher in boys than girls (p<0.001). Girls had a slightly higher school PE grade (p = 0.003) but boys reported participation in a greater number of different types of sports (p<0.001), as well as higher frequency of sports during leisure time (p<0.001) (table 1).

The two measures of early motor development, age at walking supported and age at standing unaided, were positively correlated (r = 0.54, p<0.001). Birth weight was weakly inversely associated with both age at walking supported (r = −0.04, p<0.001) and age at standing unaided (r = −0.04, p<0.001), indicating that larger birth weight infants had slightly earlier motor development. Birth season was correlated with both age at walking supported (r = 0.03, P = 0.019) and age at standing unaided (r = 0.03, p = 0.007), suggesting those born later in the year show slower infant motor development (table 2).

**Table 2:** Correlations between infant motor development and indicators of sports participation at age 14 years.

	Gestational age (weeks)	Birth weight (SD score)	Birth season	Social class	BMI at 14y (SD score)	Age at walking supported (months)	Age at standing unaided (months)	School PE grade	Frequency of sports participation	Number of different types of sports
**Gestational age (weeks)**	1.00	−0.01	0.04(**)	−0.03(**)	0.03(**)	−0.08(**)	−0.10(**)	−0.01	−0.01	0.01
**Birth weight (SD score)**	−0.01	1.00	−0.03(**)	−0.02(*)	0.11(**)	−0.04(**)	−0.04(**)	0.01	0.01	0.02(*)
**Birth season**	0.04(**)	−0.03(**)	1.00	−0.01	−0.04(**)	0.03(*)	0.03(**)	−0.08(**)	−0.02	0.00
**Social class**	−0.03(**)	−0.02(*)	−0.01	1.00	0.00	0.06(**)	0.02	−0.11(**)	−0.06(**)	−0.04(**)
**BMI at 14y (SD score)**	0.03(**)	0.11(**)	−0.04(**)	0.00	1.00	−0.03(**)	−0.04(**)	−0.11(**)	−0.02	−0.03(*)
**Age at walking supported (months)**	−0.08(**)	−0.04(**)	0.03(*)	0.06(**)	−0.03(**)	1.00	0.54(**)	−0.10(**)	−0.03(**)	−0.04(**)
**Age at standing unaided (months)**	−0.10(**)	−0.04(**)	0.03(**)	0.02	−0.04(**)	0.54(**)	1.00	−0.08(**)	−0.02	−0.03(*)
**School PE grade**	−0.01	0.01	−0.08(**)	−0.11(**)	−0.11(**)	−0.10(**)	−0.08(**)	1.00	0.30(**)	0.21(**)
**Frequency of sports participation**	−0.01	0.01	−0.02	−0.06(**)	−0.02	−0.03(**)	−0.02	0.30(**)	1.00	0.36(**)
**Number of different types of sports**	0.01	0.02(*)	0.00	−0.04(**)	−0.03(*)	−0.04(**)	−0.03(*)	0.21(**)	0.36(**)	1.00

* p<0.05.

** p<0.01 (2-tailed).

Father's social class in 1966 was positively correlated with age at walking supported (r = 0.06, p<0.001), indicating that infants in lower social class families had slower motor development. However there was no association between social class and the age at first standing unaided.

Birth weight was weakly positively correlated with the number of different types of sports activities (r = 0.02, p = 0.039) but there were no other associations between birth weight or infant growth and PE grade or frequency of sports participation. Birth season was inversely correlated with PE grade (r = −0.08, p<0.001), indicating birth later in the year was correlated to lower PE grade (table 2).

BMI at age 14 years was negatively correlated with school PE grade (r = −0.11, p<0.001) and number of different sports (r = −0.03, p = 0.001). There were no associations between BMI and frequency of sports participation at age 14 years. (table 2).

Higher school PE grade was positively correlated with increased frequency of sports participation during leisure time (r = 0.33, p<0.001) and participation in a greater number of different types of sports (r = 0.21, p<0.001). Participants who reported a higher frequency of sports also reported a greater number of different types of sports (r = 0.44, p<0.001) (table 2).

**Figure 1:**
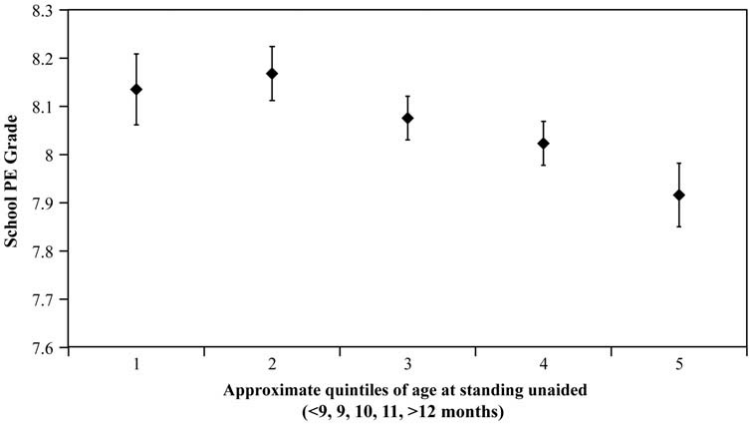
Infant motor development and school grade in physical education. School physical education grade by approximate quintiles of age at standing unaided, adjusted for sex, gestational age, birth season and father's social class category in 1966. Data are Mean and 95% C.I (n = 5,191). P for trend<0.001

For initial regression models adjusted for sex, gestational age, birth season and fathers social class; earlier infant motor development in terms of age at walking supported and age at standing unaided were both associated with higher PE grade at age 14 years (both β = −0.06, p<0.001) (table 3 and figure 1). Earlier age at walking supported was associated with a higher frequency of sports participation (β = −0.05, p = 0.024) (table 3). Similarly, age at walking supported (β = −0.04, p<0.001) and age at standing unaided (β = −0.03, p = 0.010) were associated with participation in a greater number of different types of sports at age 14 years (table 3).

**Table 3:** Associations between infant motor development and proxy indicators of physical activity at age 14 years.

	Model 1*			Model 2 †		
	β	95% CI	P	β	95% CI	P
**School PE grade**						
Age at walking supported (months)	−0.06	−0.07, −0.04	<0.001	−0.06	−0.08, −0.04	<0.001
Age at standing unaided (months)	−0.06	−0.08, −0.04	<0.001	−0.06	−0.09, −0.04	<0.001
**Frequency of sports participation**						
Age at walking supported (months)	−0.05	−0.08, −0.01	0.024	−0.04	−0.09, 0.00	0.043
Age at standing unaided (months)	−0.05	−0.09, 0.00	0.069	−0.05	−0.10, 0.00	0.082
**Number of different types of sports**						
Age at walking supported (months)	−0.04	−0.05, −0.02	<0.001	−0.03	−0.05, −0.01	0.003
Age at standing unaided (months)	−0.03	−0.05, −0.01	0.010	−0.02	−0.05, 0.00	0.091

* Model 1 adjusted for sex, gestational age, birth season and father's social class category in 1966.

† Model 2 adjusted for sex, gestational age, birth season, father's social class category in 1966, birth weight SD score and BMI SD score at age 14 years.

β Beta coefficients in this model show the change in outcome measure, for example school PE grade, per one unit change in exposure, for example 1 month earlier age at walking supported.

We thereafter examined whether these associations were independent of birth weight and BMI at age 14 years. Earlier age at walking supported and earlier age at standing unaided both remained independently associated with higher PE grade (table 2). The β-coefficients (β = −0.06, p<0.001) for these associations were virtually unchanged following the adjustments for birth weight and BMI at 14 years (table 3).

Similarly, age at walking supported remained associated with frequency of sports participation per week (β = −0.04, p = 0.043) after additional adjustments for birth weight and BMI at age 14 years (table 3). Finally age at walking supported also remained associated with participation in a greater number of different types of sports, although this association was slightly attenuated (β = −0.03, p = 0.003).

## Discussion

Our results consistently suggest that earlier infant motor development was associated with higher PE grade, higher frequency of participation in leisure time sports, and participation in a greater number of different types of sports. While these effect sizes are relatively modest, the associations were consistent between different measures of infant motor development and were independent of sex, social class, gestational age, birth weight, season of birth and BMI at age 14 years. To put these effect sizes into context there was approximately one third of a PE grade (approximately ½ SD unit) difference or one third of an extra session of sports per week between those with the earliest motor development and those at the other end of the spectrum who attained these infant motor milestones around 6 months later. In this population the effect size of a 1 month delayed motor development on school PE grade was similar in magnitude to the effect of a 1 unit increase in BMI on school PE grade (data not shown).

To the authors knowledge this is the first study investigating the influence of infant motor development on sports participation and school PE grade in adolescence. The associations observed between infant motor development and subsequent higher levels of sports participation may be a reflection of genetic and early biological influences on motor coordination and physical performance, predisposing a child to be more active in later life because of their natural aptitude. Conversely those with slower infant motor development may well have poorer motor skills and be less competent in sports and therefore potentially less likely to enjoy and participate in such activities.

Previous studies have identified associations between earlier infant motor development and improved adult physical performance [10], [12], suggesting earlier infant motor development may influence physical function in later life. Greater childhood motor skill proficiency has been associated with increased physical activity levels [4], [5] and improved aerobic fitness in adolescents [6]. Intervention studies which improve motor skills not only increase physical activity levels but improve the reported enjoyment of physical activity [7], suggesting competence or perceived competence may be important in determining physical activity levels [8], [9].

Interestingly an association has also been noted between poorer motor skills and increased BMI and waist circumference [23], although that cross-sectional study did not provide evidence for the direction of the association. However hand control and coordination in childhood has been associated with adult obesity, suggesting that neurological function in childhood may indeed influence weight status in adulthood [24].

It is likely that the timing of infant developmental milestones is partly genetically controlled, as there is variation across and between ethnic groups, even after adjusting for confounding socio-economic factors and cultural differences [25]. Early life factors are also likely to influence infant motor development, as both low birth weight and preterm delivery are associated with delayed motor development [26]. However, there is encouraging evidence that infant motor development may be modifiable. For example longer duration of breast feeding is associated with earlier infant motor development [27], [28]. Equally some milestones, such as standing with support, seem to be more influenced by care givers [29], suggesting parenting and socio-cultural factors may also influence infant motor development.

The following limitations need consideration when interpreting the findings from this study. Our measures of motor development relied on parental report rather than objective assessment, and could therefore be subject to both unintentional recall bias as well as potential deliberate reporting bias by the parents. This could be of concern if parents had differentially reported motor development, in terms of being unwilling to report delayed development, as it is possible this would lead to an overestimation of the effect size. It must also be noted that motor development was assessed at age 1 year, so some individuals with slower development would not have attained these milestones by the time of the assessment. According to WHO reference ranges above 99^th^ centile would achieve walking supported by 11.7 months and 75^th^ would achieve standing unaided by 12.2 months [30]. Therefore this study may not have captured the full range of development rates within the population, although data on at least one milestone were available for approximately 90% of the participants at age 1 year.

Although we used self reported sport participation and school PE grade, these have both been associated with overall levels of physical activity in adolescents [16], [17] and with physical activity in later life [19], suggesting that our outcome measures are likely to reflect overall levels of physical activity.

There is always the potential of residual confounding. For example, it is plausible that infant motor development is influenced by other factors in the infant environment. We adjusted our analyses for father's social class at the time of birth in 1966. However social class was measured with a four category scale, so may not fully adjust for the range of social classes. Social class is known to be associated with motor development in childhood [31], and in this study later age at walking supported was correlated with lower father's social class category. However no associations were observed between father's social class and age at standing unaided. This may well reflect the differential effects of ‘care givers’ on different motor development milestones [29], for example the age at walking supported may well be more influenced by care giver interventions, such as assistance and encouragement, whereas the age at standing unaided may be more influenced by biological development, although the magnitude of the associations between the two measures of infant motor development and physical activity outcomes in this study were consistent.

Our study has several strengths, including the large population based sample comprising more than 95% of births within the catchment area during one full year. Further, our results were remarkably consistent both in continuous and categorical analyses regardless of which exposure variable was modelled against an outcome. These associations were also robust after adjusting for confounding factors and potential mediating variables such as birth weight and BMI at age 14 years. It is therefore unlikely our results are due to chance.

It would be useful to investigate these associations in more contemporary cohorts and within different socio-cultural settings. Future research into the associations between infant motor development and later physical activity using objective measures would be particularly useful, as this may help identify whether these associations are largely mediated by sports and leisure activities, because of aptitude and enjoyment, or whether habitual physical activity levels are altered. Lastly it would also be of benefit to investigate whether the influence of infant motor development on physical activity levels extends to long term influences on body composition, obesity and metabolic risk.

Our results clearly suggest that infant motor development predicts higher sport participation. Earlier work in this cohort identified that participation in sports, at least once a week for girls and twice a week for boys at age 14 years, was associated with higher levels of physical activity at age 31 years [19]. Taken together, identifying children with slower infant motor development might help target interventions to improve motor skills with the aim of improving sports participation and overall physical activity levels later in life.
